# A Retrospective Chart Review and Infant Feeding Survey in the Irish Phenylketonuria (PKU) Population (2016–2020)

**DOI:** 10.3390/nu15153380

**Published:** 2023-07-29

**Authors:** Jane Rice, Jenny McNulty, Meabh O’Shea, Teresa Gudex, Ina Knerr

**Affiliations:** 1 National Centre for Inherited Metabolic Disorders, Children’s Health Ireland, Temple Street, D01 YC67 Dublin, Ireland; jenny.mcnulty@cuh.ie (J.M.);; 2 UCD School of Medicine, University College Dublin, Belfield, D04 V2P1 Dublin, Ireland; 3 Metabolic Health, Starship Child Health, Central Auckland, Auckland 1010, New Zealand

**Keywords:** Phenylketonuria, PKU, breastfeeding, complementary feeding, education

## Abstract

Phenylketonuria (PKU) is an inherited disorder of protein metabolism. It is generally treated using dietary management with limited intake of phenylalanine (Phe). Partial breastfeeding (BF) is encouraged among mothers of infants with PKU, together with a Phe-free mixture of synthetic amino acids. Our aim was to describe our current BF rates and complementary feeding practices, as well as examining parental experiences of infant feeding. The objective was to better understand the challenges faced by families so that improvements can be made to clinical care. A chart review was carried out on 39 PKU patients, examining the BF rate and duration, use of second stage synthetic protein (SP), and average complementary feeding age. A parental questionnaire on complementary feeding and BF experience was designed: 26% of babies were partially breastfed at three months of age; 70% of mums would like to have breastfed for longer and cited PKU as a reason for stopping; 52% of parents reported challenges during the complementary feeding process including food refusal, protein calculation, and anxiety around maintaining good Phe levels. Suggestions to improve BF continuation and duration include active promotion of the benefits and suitability, access to lactation consultant, and peer support. The delay in introducing a second stage SP may contribute to long-term bottle use for SP. Improved patient education, written resources, and support is necessary to improve food choices and long-term acceptance of SP.

## 1. Introduction

Phenylketonuria (PKU) is an autosomal recessive inherited disorder of phenylalanine metabolism caused by pathological variants in the phenylalanine hydroxylase (*PAH*) gene, which results in a deficiency of phenylalanine hydroxylase (PAH) [1]. This causes an accumulation of phenylalanine (Phe) in the blood and brain. If left untreated it can lead to a variety of clinical manifestations, including acquired microcephaly, epilepsy, severe cognitive impairment, behavioural disorders, and seizures [2].

In Ireland, PKU has been diagnosed through the national newborn bloodspot screening programme (NNBSP) since 1966, between 72 and 120 h after birth [3]. Treatment should begin as early as possible, ideally before 10 days of life and generally consists of a lifelong restriction of Phe intake, supplemented with a low-Phe or Phe-free synthetic amino acid formula [4]. Upon diagnosis, patients are generally admitted to our hospital for initiation of dietary treatment and parental education. Frequent blood Phe monitoring is needed to guide dietary treatment, particularly during the first years of life [4]. 

Breastfeeding (BF) has been reported to have many benefits including, e.g., reduced risk of infant mortality, lower rate of obesity, malocclusion, otitis media, or asthma, and promotion of better intellectual development with higher intelligence quotients [5]. Benefits to BF mothers may include lower rates of breast cancer, ovarian cancer, type II diabetes, and postpartum depression [5]. 

Infants with PKU can be partially breastfed, allowing exposure to the benefits of breast milk [4]. Acceptable blood Phe control and growth are possible with its use [6]. However, breastfeeding rates and duration of feeding in infants with PKU remain lower than in the general population [7]. There is limited research investigating the maternal experience of BF an infant with PKU. 

Complementary feeding is the process of introducing solid or more textured foods into an infant’s liquid-based diet. This should begin at around 6 months of age, to ensure acceptance of new tastes and textures, and include a wide variety of foods [8]. This is of particular importance in PKU, as there are many dietary restrictions. A more concentrated SP is usually introduced at 6 months to ensure adequate total protein intake without affecting appetite for solid food [9]. 

The aim of our study was to describe the infant feeding and complementary feeding practices in an Irish patient cohort. Our survey also aimed to explore mothers of infants with PKU and their experience of BF. We wanted to determine the factors which affected early BF cessation. We also aimed to understand the challenges faced by parents whilst complementary feeding a child with PKU. This information will be used to improve our service delivery to patients.

## 2. Materials and Methods

### 2.1. Data Collection

#### 2.1.1. Retrospective Chart Review 

A retrospective review of dietetic patient records was performed at the National Centre for Inherited Metabolic Disorders (NCIMD) in Children’s Health Ireland (CHI) at Temple Street. The NCIMD provides metabolic care to all infants diagnosed with PKU in the Republic of Ireland. A retrospective chart review was carried out on all PKU infants with one complete year of data between August 2016 and January 2020. Our inclusion criteria were: PKU diagnosis, genetically confirmed, diagnosed by NNBSP, requiring dietary treatment, consent. Exclusion criteria were: lack of genetically confirmed PKU diagnosis or lack of consent.

Data collected included feeding method at: diagnosis, discharge from CHI, 3 months, 6 months, and 12 months of age, duration of BF, and BF modalities. We also collected data on gender, age at diagnosis, Phe level at diagnosis, Phe levels between 3 and 52 weeks of age, age of complementary feeding, and the timing of introduction of second stage SP. Ethical approval was obtained from the Research Ethics Committee, CHI, at Temple Street. 

#### 2.1.2. Parental Survey 

A parental questionnaire (Supplementary Materials) was designed using the platform ‘Survey Monkey’. Parents of patients identified through the chart review were invited to participate in the survey exploring maternal experiences of BF infants with PKU, as well as their experience of complementary feeding. Informed consent was obtained from any parent willing to participate. The survey questionnaire included 61 questions, with key question domains including ethnicity, previous experience of BF, impact of diagnosis of PKU on BF, facilities to BF/express, access to equipment, support/encouragement to restart BF in hospital, BF support at home, BF difficulties, duration of BF, and reasons for stopping. 

The second main section focused on the complementary feeding experience encompassing first foods, exposure to inappropriate complementary feeding foods, use of shop bought versus homemade prepared foods, information provided on complementary feeding, challenges faced during the complementary feeding process, main sources of stress during this period, and the use of bottle or beakers.

The full questionnaire is available as on online supplement. Response options included both forced choice and open-ended questions. The survey was developed by metabolic dietitians and pilot testing of the questionnaire was performed with a PKU parent who was not included in this study to ensure the questions were clear. 

### 2.2. Statistical Analysis

All data were analysed using Microsoft Excel, Version 2013, and GraphPad Prism. All data are reported as means, medians, SD, or numbers (%) unless otherwise indicated. The differences between the Phe levels in the BF and non-BF groups were determined by Mann–Whitney U test. Significant values were considered for *p* < 0.05.

## 3. Results

### 3.1. Retrospective Chart Review

#### 3.1.1. Demographic 

Thirty-nine PKU infants (35.9% female, *n* = 15) were identified as requiring dietary treatment during the study period. The diet commenced at a mean age of 8.3 days +/−2.2 days. Ninety-seven percent (*n* = 38) of mothers were Irish. Seventy-nine percent (*n* = 31) of patients had been admitted to hospital for initiation of dietary treatment. Of the six patients not admitted, three had siblings with PKU, two had blood Phe levels < 600 µmol/L—indicative of early diagnosis on NNBSP—and one was readmitted with their mother to the maternity hospital due to post-partum complications.

#### 3.1.2. Feeding Method

**Figure 1:**
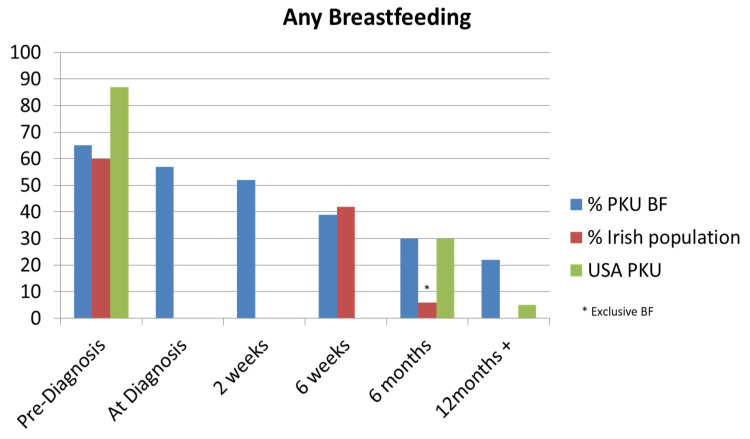
Comparison of any breastfeeding in the Irish Phenylketonuria (PKU) cohort with the Irish general population [10,11,12] and an American PKU study [13].

From our patient cohort, 41% (*n* = 16) were BF at diagnosis. In our BF patient group, the mean duration of BF was 30.2 weeks (median 23.5 weeks; range 0.8–97.5 weeks). Partial BF was the sole source of natural protein intake for a mean duration of 15.4 weeks (median 15.5 weeks; range 0.8–41 weeks). A total of 31% of patients were using breastmilk as a source of natural protein at 4 weeks of age, 26% at 3 months of age, and 23% at 9 months of age. We compared our BF results with the general Irish population and an American PKU study as depicted by Figure 1.

The traditional method of prescribed amount of Phe-free infant formula followed by breastfeeds to appetite was advised in 94% of cases in our cohort. One patient followed a method of alternating between Phe-free infant formula and BF.

**Figure 2:**
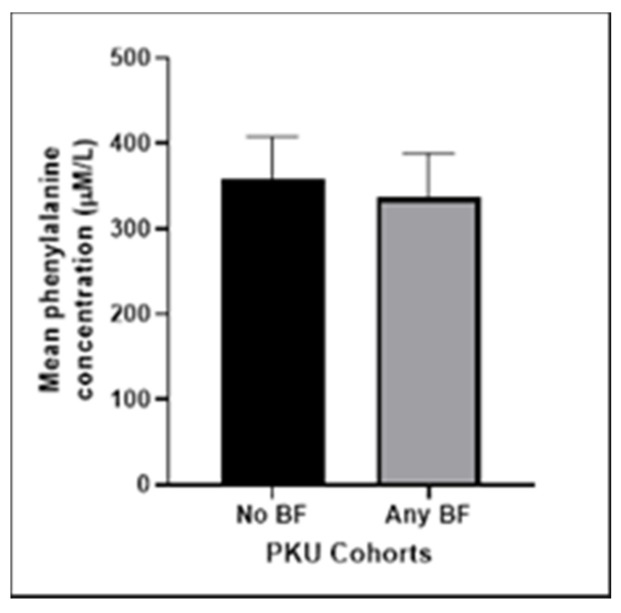
Mean phenylalanine concentration (μmol/L) of our PKU cohort comparing the non-breastfeeding group with those who did some breastfeeding post diagnosis (*p* = 0.0705).

We compared the mean Phe results of the BF and non-BF groups. The BF group had marginally better metabolic control compared to the non-BF group as depicted by Figure 2. However, the difference between the means did not reach statistical significance (*p* = 0.0705).

#### 3.1.3. Complementary Feeding

The mean age at which complementary feeding was commenced was 21.2 weeks (median 22 weeks; range 14.5–26.5 weeks). One infant was introduced to complementary feeding too early at 14.5 weeks of age. All patients were offered and attended an appointment to discuss complementary feeding before commencing. A second stage SP was introduced at a mean age of 33 weeks. The majority of patients were given a paste-style SP as their first option. 

### 3.2. Parental Survey Results

#### 3.2.1. Breastfeeding Responses

The response rate to the parental survey was 23/39 (59%). Of the participants, 43% reported having previous experience with BF their older children. At the time of diagnosis, 65% (*n* = 15) of respondents were BF mothers. However, five mothers stopped BF due to their child’s PKU diagnosis. Feedback provided by the respondents, as shown in Table 1, revealed the reasons why they continued or discontinued BF. Reasons cited for discontinuing included wanting to feel in control of the amount of natural protein being given, while others found combination feeding burdensome. Some mothers reported feeling stressed due to their inexperience, coupled with the stress of the diagnosis. 

**Figure 3:**
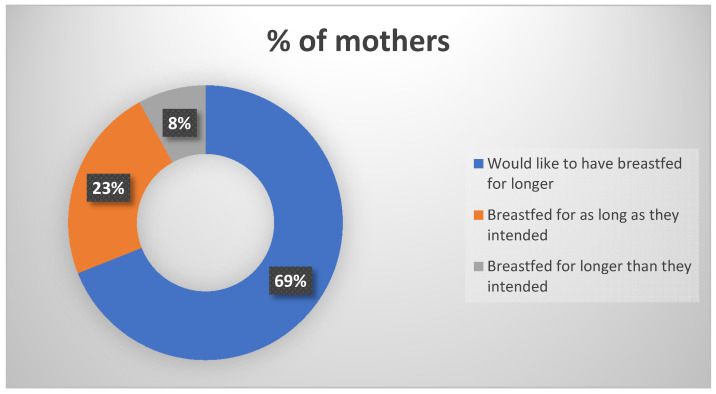
Maternal feeling regarding the duration of their breastfeeding journey.

The majority of BF respondents, i.e., 77% (*n* = 10), reported that hospital facilities were supportive of BF. However, 23% (*n* = 3) reported a lack of privacy to express, with one reporting a delay in getting a BF pump. A total of 85% (*n* = 11) of respondents reported BF difficulties, including poor supply, breast and nipple pain, poor attachment, blocked duct, delay in feeding due to premature birth, tongue tie, complications with oversupply as a result of tandem feeding with Phe-free formula, and lack of BF support in the time between PKU diagnosis and admission to hospital. Among respondents, 69% (*n* = 9) sought out BF advice or support. Their main source of support included metabolic dietitians, husband/partner, midwives, lactation consultant, other mothers who had breastfed their children with PKU, and online support. A total of 89% (*n* = 8) of respondents described this support as either extremely or very helpful, with 11% (*n* = 1) describing it as somewhat helpful. Figure 3 illustrates the responses from our mothers in relation to their breastfeeding journey 

PKU-related reasons for interrupting BF included PKU diagnosis, poor supply, nipple confusion, maternal health/stress, quantifying natural protein intake, convenience, and pressure from the metabolic team. 

#### 3.2.2. Complementary Feeding Responses

The responses to questions on complementary feeding were answered by 91% (21/23). A total of 71% (*n* = 15) respondents started with puree vegetables as the first food offered. Among the respondents, 25% exposed their offspring to one or more inappropriate foods including crisps, sweets, biscuits, sugary cereals, and ice cream before the age of 1 year. Ninety-five percent (*n* = 20) of respondents mostly prepared homemade baby foods, and 62% (*n* = 13) sometimes used pre-made baby foods. Elements of a baby-led approach to complementary feeding were incorporated by 90% (*n* = 19). With regards to complementary feeding information, 95% (*n* = 20) of respondents were satisfied with the standard of information provided by the metabolic team. Over half of the respondents (*n* = 11) reported challenges during the complementary feeding process. Stress during this period was reported by 81% of respondents. The challenges encountered are listed in Table 2 (*n* = 17).

62% (*n* = 13) of respondents were still using a bottle for the SP beyond 1 year of age and 43% (*n* = 9) were still using a bottle for the synthetic protein beyond 18 months of age. This is despite the majority of parents (62%, *n* = 13) introducing a sippy cup at around 6 months of age and despite advice to wean off the bottle around the age of 12 months.

## 4. Discussion

In our chart review, we found dietary treatment was started at a mean age of 8.3 days. This aligns with the recommendation in the European PKU guidelines that treatment should start as soon as possible, ideally before 10 days of age [4]. Our centre’s standard procedure is to admit new patients diagnosed by NNBSP for further diagnostic workup and commencement of treatment with MDT input. Exceptions are made for siblings of known PKU patients. We explored parents’ experiences of their hospital admission through a parental survey questionnaire in our cohort of PKU patients. Our findings showed that the PKU population exhibited lower rates of BF compared to the general Irish population in relation to offspring aged 3 months (26% vs. 35.4%) [10]. However, at 6 months of age, the PKU group exhibited higher rates of BF than the general population [11]. It is worth noting that published BF figures in Ireland are limited, and our figures compared mothers who were partially BF with those who were exclusively BF at the same age [12]. A similar decline in BF was observed after PKU diagnosis in an American–Canadian study, but more than 50% of this population were still BF when the offspring was aged 6 months [14]. A European study showed a more similar value of 30% BF rate with offspring beyond 6 months of age [15]. This variability in both the rates of BF continuation post diagnosis and duration highlights the complexity of supporting mothers through the BF journey. 

Our PKU BF duration was also compared to an audit completed in our centre in 2006–2007, which showed an improvement in mean duration from 15.8 weeks to 30.2 weeks. This improvement is encouraging for our management of PKU. We cannot influence the BF initiation rates; however, our role is key in supporting continuation of BF beyond the diagnosis of PKU. Our finding would suggest that additional metabolic multidisciplinary (MDT) support is needed to overcome the stress of combined feeding and that associated with dealing with a new diagnosis. Reassurance and promotion of the benefits of BF, specifically in relation to PKU, appears to have influenced some mothers’ decision to persist with BF. 

Our results suggest that metabolic control is as good in BF patients as it is in those who are formula-fed, although this finding did not reach statistical significance. A similar outcome is described in Kose et al., where Phe levels in a cohort of 26 infants were found to be better controlled when compared to the bottle-fed group [16]. These findings are worth highlighting to counter the parental and medical belief that it is more difficult to control blood Phe levels when BF. Breast milk is the best source of nutrition for infants, and this is especially important for babies affected by PKU, as breast milk contains lower levels of Phe than formula [4]. 

Complementary feeding amongst our PKU cohort meets current general population guidelines advising commencement at around 6 months of age, but no earlier than 17 weeks of age [8]. This should follow a similar approach to the general guidelines with a gradual introduction of semi-solid and solid foods, beginning with protein-free vegetables and fruit. Only one infant was introduced to complementary feeding too early, at 14.5 weeks of age, despite all patients being offered a clinic appointment prior to starting complementary feeding. A key difference in the PKU approach to complementary feeding is the need for a transition to a second stage SP. This should ideally happen upon commencement of complementary feeding to help reduce fluid volumes and increase appetite for solids [9]. However, we found that a second stage SP was introduced at a mean age of 33 weeks. The majority of patients were given a paste-style SP as their first option. One of the main reasons for delayed introduction of a second stage SP was the overwhelming number of dietary components which parents are required to balance during this period. 

The parental survey results showed that the majority of respondents experienced BF difficulties and sought BF advice or support. Their primary sources of support included lactation consultants, partners, midwives, other mothers who had breastfed their children with PKU, metabolic dietitians, and online support. Despite the challenges faced, the majority of respondents would like to have breastfed for longer. In order to enhance our breastfeeding rates, it is essential that we carefully examine all the reasons provided for discontinuing breastfeeding. This is particularly crucial, since 69% of the respondents expressed a desire for a longer breastfeeding duration. We also noted the impact of the introduction of protein containing foods on continued BF. It is vital to encourage BF throughout the complementary feeding stage while discouraging the perception of the latter as a time to begin weaning from the breast. 

BF rates among mothers whose babies are affected by PKU can be improved through education and support. Suggestions to increase BF continuation rates and duration include active promotion of the benefits and suitability, collective efforts of all MDT members in aiding, alleviation of maternal concerns, access to lactation consultants, and peer support. Connecting mothers with other mothers who have successfully breastfed their baby with PKU can be a valuable source of support. This can be done through support groups, online forums, or other types of peer support networks. Our new national children’s hospital, which is due to open in 2024, will address issues some of our mothers faced concerning inadequate privacy to express breastmilk. It will provide individual rooms for patients and their parents. This aligns with one of the objectives of the WHO/UNICEF Baby-Friendly Initiative [17].

A recurring issue reported by new mothers since our study is the inadequate provision of feeding information from local maternity hospitals during the period between PKU diagnosis and admission to the metabolic centre. This lack of information is a significant source of stress for families and may impact the mother’s decision to continue BF. Several mothers are unfamiliar with PKU and its management, a phenomenon which highlights the importance of education around the condition. Providing such an education could help mothers feel more empowered and confident in their ability to breastfeed their baby with PKU. Since 2019, pharmacotherapy for PKU in the form of sapropterin has been offered to all infants diagnosed with PKU by NNBSP in Ireland. This means that they can continue breastfeeding for the duration of the 24 h sapropterin trial. This factor may have a positive impact on BF continuation and warrants further investigation.

Healthcare providers play a critical role in supporting mothers who want to breastfeed their baby affected by PKU. This includes providing guidance on BF techniques, monitoring the baby’s growth and development, and helping to manage any challenges that arise. We acknowledge the importance of additional education and training regarding breastfeeding assistance for the entire MDT. While BF is the ideal choice for babies with PKU, it may not always be possible or practical for some mothers. Providing information on alternative feeding options, such as expressing breast milk, can help ensure that all babies with PKU receive the proper nutrition they require. 

Our survey revealed that the parents in our cohort are establishing healthy eating habits from the early stages, with 71% of respondents initiating their babies’ solid food journey with pureed vegetables. A total of 90% of the parents reported the inclusion of elements of “baby-led” feeding into their child’s complementary feeding journey. This was in agreement with a study by Evans et al. which showed an appropriate progression to solid foods and feeding skills among infants with PKU when compared to a non-PKU control [18]. 

Furthermore, a very high proportion of respondents prepared homemade meals each day. This is in contrast to the findings of a study conducted among the general population in the United Kingdom which reported that 72% of seven-to-nine-month-olds and two thirds (67%) of ten-to-eleven-month-olds had eaten a commercial baby or toddler meal as their main meal of the day [19]. This may reflect our dietetic efforts to promote home-prepared meals as a means of building healthy dietary habits from the earliest stages. In our experience, building healthy dietary habits early in life and with MDT input is beneficial for long-term compliance given the complexity of PKU management [20]. 

Over half of the respondents in our survey reported challenges during the complementary feeding process, such as food refusal and difficulty calculating the protein content of foods. Despite attending regular appointments to discuss this process, some parents still used inappropriate foods for their child. Delayed introduction of a second stage SP may contribute to long-term bottle use for SP. Therefore, it is important to improve education and support to encourage healthy food choices and address the challenges around long-term acceptance of SP. 

Since conducting our study, we have made improvements to our complementary feeding resources, despite 95% of respondents being satisfied with the standard of information they received. Our resources now include troubleshooting advice, practical recipes for integrating protein exchanges, visual guides, and healthy eating guidelines. Previously, the parents may have seen a different dietitian at each clinic visit. We have changed our practice to assign a lead dietitian to guide parents through each stage of complementary feeding. This should promote consistency and aid the introduction of second stage SP to help reduce long-term reliance on bottles. We recognise the importance of synthetic protein as a crucial part of PKU treatment and maintaining good metabolic control. To support parents struggling to get their children to take SP we collaborate closely with our MDT, particularly our psychologists and nursing staff; our patients also underwent regular medical reviews. We understand that this can be a challenging aspect of treatment, and we aim to provide families with the necessary resources and support to promote successful adherence. We also acknowledge that PKU is a well studied condition, and that further challenges may arise for patients affected by other inborn disorders of protein metabolism who may be at risk of acute metabolic crises. However, further efforts should be made to support partial BF where medically possible.

Our research was the first to examine BF rates among the Irish PKU population. We also examined the barriers, as well as solutions, to the preservation of BF through the parental survey. This has provided unique insights into the influence of the messages around BF at the time of PKU diagnosis. Our analysis on the stable Phe control in the BF group further supports the recommendation of continued BF. Our complementary feeding section of the parental survey was the first of its kind. It has provided us with vital feedback on the daily challenges, unique to PKU, that parents face. This insight has already changed our practices and our educational material to lessen the burden of care for parents. 

Some limitations of our study include the small patient numbers, and the fact that demographic data on the parent groups in terms of age, education, marital status, and employment were not considered. We did not examine the genotypes or the Phe level of our patients at diagnosis. These may have had an impact on BF duration and continuation. Our survey was not validated, but we did engage a parent who had previously BF to review it prior to its implementation. We had hoped to conduct a semi-structured interview, which may have revealed more specific details about the challenges faced by parents. However, this was not possible due to the impact of the COVID-19 pandemic on staffing and clinic visits within our service. 

## 5. Conclusions

BF rates are increasing in the Irish cohort of infants diagnosed with PKU. However, barriers to continuation of BF exist and are more complex in PKU-affected cohorts. Our study has provided insight into the parental experiences surrounding BF and will improve the quality of care needed to support affected families. A multifaceted approach that includes education, support, and flexibility in feeding options is required. By working together with healthcare providers, peer support networks, and other resources, mothers can feel confident in their ability to provide their baby with the best possible start in relation to infant feeding. The introduction of complementary feeding is happening at an appropriate time for most of the patients diagnosed with PKU in the Irish cohort. However, more education and support is required to minimise parental stress and anxiety throughout this journey. 

## Figures and Tables

**Table 1:** Parental reports of the effect of a Phenylketonuria diagnosis on breastfeeding continuation.

**Benefits of Breastfeeding**
*“I felt at the time that I wanted to give my daughter the best possible start and thought the goodness of breast milk was better than formula”.* *“I had wanted to exclusively breastfeed anyway but when he was diagnosed I wanted prevent illnesses as much as possible so his Phe level wouldn’t increase”.* *“Terrifying to think my milk was harming baby but when assured formula would have shown same results decided to continue, secretly hoping deep down my milk would adapt to babies needs as it’s said to when they have a cold etc”.*
**Control/Accuracy/Precision**
*“Having to introduce a formula before breastfeeding, took away from breast only feeding. I did continue for approx.10 weeks, but found, PKU formula, breast and normal formula, very hard work to manage all”.*
**Overwhelming**
*“My baby was born 5 weeks premature, in SCBU for a week on CPAP, all prior to being diagnosed with PKU. I found it all overwhelming and decided to formula feed after a couple of weeks of feeding my baby expressed breast milk”.*
**Lack of Experience**
*“I was a first time mother, breast feeding can be stressful anyway but I found my baby was extremely stressed moving between the breast feeding and formula within the one feed. I was also dealing with my first baby and the worry of the diagnosis. A few years down the road and having breastfed my second child (without PKU) I think I would be more confident with breastfeeding should I have another child with PKU, having experience with breastfeeding and also PKU”.*

**Table 2:** Challenges reported by parents during the complementary feeding process.

Calculating the protein content in food Knowing how to count leftover portions Juggling spoon feeds and SP Fussy eating Managing normal baby behaviours like food refusal Educating family members/other caregivers Wrong foods given Preparation of food, always having food with them Travel/planning ahead Reflux—food reactions/intolerances Worrying about Phe levels going in/out range

## Data Availability

Not applicable.

## Supplementary Materials

The following supporting information can be downloaded at: https://www.mdpi.com/article/10.3390/nu15153380/s1.

## References

[1] Elhawary N.A., AlJahdali I.A., Abumansour I.S., Elhawary E.N., Gaboon N., Dandini M., Madkhali A., Alosaimi W., Alzahrani A., Aljohani F. (2022). Genetic etiology and clinical challenges of phenylketonuria. Hum. Genom..

[2] Ashe K., Kelso W., Farrand S., Panetta J., Fazio T., De Jong G., Walterfang M. (2019). Psychiatric and Cognitive Aspects of Phenylketonuria: The Limitations of Diet and Promise of New Treatments. Front. Psychiatry.

[3] HSE. https://www.hse.ie/eng/health/child/newbornscreening/newbornbloodspotscreening/information-for-professionals/a-practical-guide-to-newborn-bloodspot-screening-in-ireland.pdf.

[4] Van Wegberg A.M.J., Macdonald A., Ahring K., BéLanger-Quintana A., Blau N., Bosch A.M., Burlina A., Campistol J., Feillet F., Giżewska M. (2017). The complete European guidelines on Phenylketonuria: Diagnosis and treatment. Orphanet. J. Rare Dis..

[5] Grummer-Strawn L.M., Rollins N. (2015). Summarising the health effects of breastfeeding. Acta Paediatr..

[6] van Rijn M., Bekhof J., Dijkstra T., Smit P.G.P.A., Moddermam P., van Spronsen F.J. (2003). A different approach to breast-feeding of the infant with phenylketonuria. Eur. J. Pediatr..

[7] Zuvadelli J., Paci S., Salvatici E., Giorgetti F., Cefalo G., Dionigi A.R., Rovelli V., Banderali G. (2022). Breastfeeding in Phenylketonuria: Changing Modalities, Changing Perspectives. Nutrients.

[8] Fewtrell M., Bronsky J., Campoy C., Domellöf M., Embleton N., Mis N.F., Hojsak I., Hulst J.M., Indrio F., Lapillonne A. (2017). Complementary Feeding: A Position Paper by the European Society for Paediatric Gastroenterology, Hepatology, and Nutrition (ESPGHAN) Committee on Nutrition. J. Pediatr. Gastroenterol. Nutr..

[9] MacDonald A., van Wegberg A.M.J., Ahring K., Beblo S., Bélanger-Quintana A., Burlina A., Campistol J., Coşkun T., Feillet F., Giżewska M. (2020). PKU dietary handbook to accompany PKU guidelines. Orphanet J. Rare Dis..

[10] Healthcare Pricing Office. https://www.hpo.ie/latest_hipe_nprs_reports/NPRS_2019/Perinatal_Statistics_Report_2019.pdf.

[11] HSE. https://www.hse.ie/eng/services/publications/performancereports/management-data-report-june-2020.pdf.

[12] Layte R., McCrory C. (2015). Growing Up in Ireland: Maternal Health Behaviours and Child Growth in Infancy, Dublin: Stationery Office/Department of Children and Youth Affairs. https://www.esri.ie/publications/growing-up-in-ireland-maternal-health-behaviours-and-child-growth-in-infancy.

[13] Banta-Wright S.A., Shelton K.C., Lowe N.D., Knafl K.A., Houck G.M. (2011). Breast-feeding Success Among Infants with Phenylketonuria. J. Pediatr. Nurs..

[14] Banta-Wright S.A., Press N., Knafl K.A., Steiner R.D., Houck G.M. (2014). Breastfeeding Infants with Phenylketonuria in the United States and Canada. Breastfeed. Med..

[15] Pinto A., Adams S., Ahring K., Allen H., Almeida M., Garcia-Arenas D., Arslan N., Assoun M., Altınok Y.A., Barrio-Carreras D. (2018). Early feeding practices in infants with phenylketonuria across Europe. Mol. Genet. Metab. Rep..

[16] Kose E., Aksoy B., Kuyum P., Tuncer N., Arslan N., Ozturk Y. (2018). The Effects of Breastfeeding in Infants with Phenylketonuria. J. Pediatr. Nurs..

[17] Unicef, UK, Baby-Friendly Initiative Standards. https://www.unicef.org.uk/babyfriendly/wp-content/uploads/sites/2/2014/02/Guide-to-the-Unicef-UK-Baby-Friendly-Initiative-Standards.pdf.

[18] Evans S., Daly A., Wildgoose J., Cochrane B., Chahal S., Ashmore C., Loveridge N., MacDonald A. (2019). How Does Feeding Development and Progression onto Solid Foods in PKU Compare with Non-PKU Children During Weaning?. Nutrients.

[19] Lennox A., Sommerville J., Ong K., Henderson H., Allen R. (2011). Diet and Nutrition Survey of Infants and Young Children.

[20] Clark A., Merrigan C., Crushell E., Hughes J., Knerr I., Monavari A.A., Treacy E., Coughlan A. (2019). Ten-year retrospective review (2003–2013) of 56 inpatient admissions to stabilize elevated phenylalanine levels. JIMD Rep..

