# Supplementary material for: A Retrospective Chart Review and Infant Feeding Survey in the Irish Phenylketonuria (PKU) Population (2016–2020)
Source: Nutrients. 2023 Jul 29;15(15):3380. doi: 10.3390/nu15153380 (PMC10421512; doi:10.3390/nu15153380)
Supplement: Supplementary file 1 [file nutrients-15-03380-s001.zip › nutrients-2490540-supplementary.pdf]

Questions for phone survey

- Hi, can I speak to X please? This is X calling from the Metabolic Unit,

I'm getting in touch regarding a study you agreed to participate in recently called

'A retrospective review of infant feeding and weaning practices in the Irish PKU cohort 2016-2019'.

- As you recall, this study involves us, the Metabolic Team,

calling participants to discuss their recent infant feeding and weaning experiences.

Do you have a moment to talk now?

There are a total of 30 questions in the survey and it should take no more than 15- 45 minutes, depending on your feeding experiences.

- Ok great, just to give you a bit of background on the study,

we are looking to gain information on infant feeding and weaning so that we can improve

the experience for mothers with babies with a diagnosis of PKU.

Do you have any questions before I begin?

You can stop me at any stage if you have any queries.

1. What ethnicity do you (mum) identify as?
   - □ Irish
   - □ United Kingdom
   - □ North America
   - □ South America
   - □ Central European
   - □ Eastern European
   - □ Scandinavian
   - □ Asian
   - □ African
   - □ Australia/New Zealand
   - □ Russian
   - □ Other

2. Thinking back to before your baby was born, how did you plan to feed them? (Tick one)
   - □ Breast
   - □ Formula
   - □ Combination of breast and Formula
   - □ I hadn't decided

2a. Did you have any experience of breastfeeding with previous children?
- □ Yes
- □ No

2b. Was it a positive or negative experience?
- □ Positive

□ Negative

3. How was your baby fed at the time they were diagnosed with PKU (before they had started any PKU formula). (Tick one)
   - □ Breast
   - □ Formula
   - □ Combination of breast and Formula
   - □ Expressing breast milk
   - □ My baby was not feeding
   - □ Can't remember

<u>If the answer to question 3 was formula only skip to question 18, otherwise continue:</u>

BREASTFEEDING SECTION

3a. Did your baby's PKU diagnosis influence you in making your decision to breastfeed or express milk?

□ Yes
□ No

3b. If yes, did this influence cause you to:

i. Continue, or
ii. Discontinue

4. Were you ever given conflicting advice by health professionals for example a GP, Public Health Nurse (PHN), midwife, member of the metabolic team about whether your child could be breast fed?
   - □ Yes
   - □ No

*If do not identify party, ask

5. Who or what source did you find gave the most helpful information about feeding, since your baby was diagnosed with PKU? (open text)

6. When your baby was admitted to hospital following their diagnosis with PKU, did you have enough support with breastfeeding (e.g. professional support, written information, visual resources?
   - □ Yes
   - □ No

6a. If answered no to above: Can you elaborate or tell me more about this? (open text)

6b. Were the facilities in Temple Street supportive of breastfeeding e.g. comfortable, private, easily accessible breastfeeding equipment,
access to catering facilities?

□ Yes
□ No

6c. If answered no to above: Can you elaborate or tell me more about this? (open text)

7. Were you given advice about how to express your breast milk during your child's hospital admission?

□ Yes
□ No

7a. Were you given a breast pump when your baby was admitted to hospital after their diagnosis?

□ Yes
□ No

7b. Were you given instructions on how to use the breast pump e.g. written instructions/demonstration?

□ Yes
□ No

7c. Did you feel that those instructions were useful?

□ Yes
□ No

7d. Did you hand express at any stage?

□ Yes
□ No

If answered no to 7c

7c. Were you ever shown how to hand express?

□ Yes
□ No

8. When reintroducing natural protein with the phe-free formula, were you ever influenced not to use breastmilk?

□ Yes
□ No

8a. If answered yes to above:

Can you elaborate or tell me more about this?

9. Did you experience any problems breast feeding your baby at any stage?
(e.g. breast pain, nipple pain, mastitis, poor supply, poor latching, unsettled baby, difficulties relating to your recovery after birth)

- □ Yes
- □ No

If yes, can you elaborate or tell me more about this?

9a. If yes to above, did you seek out any breast feeding advice or support?

- □ Yes
- □ No

9b. If yes to 9a: Where did you look for advice or support e.g. internet, breastfeeding group, lactation consultants, PKU Mum who previously breastfed etc?
(Free text, any sources of advice/support – ask which was the most helpful)

9c. Please tell us how helpful the main source was:

□ Excellent □ Very Good □ Satisfactory □ Poor □ Very Poor

9d. If no to 9a – Were there any reasons why you didn't seek advice?

9e. Thinking back now, was there any additional support or information which you would have found helpful?

10. How do you feel about your experience breast feeding your baby?

Prompt if necessary – positive/negative experience – what would be the main reason you found it to be an X experience?

11. Did you give your baby a combination of standard infant formula e.g. SMA/Aptamil/Cow+Gate and breast milk at any stage?

- ◻ Yes
- ◻ No

11a. If yes, what was your main reason for doing this?

12. Did you ever feel influenced by a health professional for example a GP, PHN, midwife, member of the metabolic team or other to stop breast feeding your child at any time?

- ◻ Yes
- ◻ No

12a. If answered yes to 12: Can you tell me more about this?

13. How old was your baby when you stopped breast feeding them? (x age)
14. What was your reason(s) for stopping breastfeeding?

15. Which of the following best describes your experience breastfeeding your baby?

◻ I would like to have breastfed for longer
◻ I breastfed for as long as I had intended
◻ I have breastfed for longer than I had intended

16. What, if anything, would have helped you to continue breastfeeding for longer?

17. Did you feel pressure to continue breastfeeding at any stage?

- ◻ Yes
- ◻ No

If yes, please elaborate

This concludes our section on breastfeeding, is there anything else you would like to talk to me about with regards to breastfeeding

WEANING SECTION (ask all parents)

18. How old was your child when they started weaning onto solid foods/spoon feeds? (x age) _______
19. How did you decide when to start weaning your baby? (open text)

20. What was the first solid food your baby was given? (open text)

21. At any stage under 1 year of age did your baby eat or drink the following foods? (yes/no/can't remember to the below list of foods)

	Yes	No	Can't remember
A Regular yoghurt (clarify not low protein/dairy free)			
B Eggs			
C Nuts or seeds e.g nut butters			
D Regular cheese (clarify not low protein/vegan)			
E Regular cow's milk (clarify not low protein)			
F Potato waffles or hash browns or croquettes			
G Chips or wedges			
H Crisps			
I Juice e.g. cordial, squash, fruit juice			
J Fizzy drinks			
K Sweets or chocolate or lollipops (including low protein chocolate)			
L Biscuits or cake (including "baby" biscuits e.g.rusks and low protein biscuits/cake)			
M Sugar sweetened cereals e.g. Low protein loops, coco pops, crunchy nut, cookie crunch, frosties			
N Rice Cakes (including baby rice cakes)			
O Vegetable fingers			
P Ice cream			

22. When weaning, how often did you give your baby each of the following:

a.) Store-bought baby foods (NOT prescription foods): □Mostly □Often □Sometimes □Never

b.) Home-made/home prepared foods: □Mostly □Often □Sometimes □Never

22b. Can you remember what influenced you in making these choices?

23. Thinking about the information you received about weaning your baby, who or what was your main source of information?

24. Did you have an outpatient appointment to learn about weaning before your baby started weaning?

- □ Yes
- □ No

25. Were you satisfied with the information you received about weaning?

- □ Yes
- □ No

26. Did you incorporate any elements of a baby led approach to weaning when weaning your child? E.g. allowing your baby to feed themselves after 6 months by offering some soft finger foods

- □ Yes
- □ No

27. Did you encounter any challenges during the weaning process?

- □ Yes
- □ No

27a. If answered yes to above, can you tell me more about this?

28. Thinking back now, was there any additional support or information about weaning which you would have found helpful?

29. How did you feel about your experience weaning your child?

30. The weaning process can be a challenging time. Did you feel stress during this period?

□ Yes
□ No

30a If yes what were the main sources of stress: e.g. getting exchanges in, food refusals, juggling different elements of the diet

31. How old was your child when you introduced a beaker/sippy cup? (x Age) _______

32. What age did they stop taking their synthetic protein from a bottle completely? (By this we mean they took their synthetic as a spoonable paste like first spoon or gel/squeezie, or as liquid in a beaker/sippy cup)

□Age _______
□Still taking from a bottle currently
